# Melatonin improves the first cleavage of parthenogenetic embryos from vitrified–warmed mouse oocytes potentially by promoting cell cycle progression

**DOI:** 10.1186/s40104-021-00605-y

**Published:** 2021-07-16

**Authors:** Bo Pan, Izhar Hyder Qazi, Shichao Guo, Jingyu Yang, Jianpeng Qin, Tianyi Lv, Shengqin Zang, Yan Zhang, Changjun Zeng, Qingyong Meng, Hongbing Han, Guangbin Zhou

**Affiliations:** 1 Farm Animal Genetic Resources Exploration and Innovation Key Laboratory of Sichuan Province, College of Animal Science and Technology, Sichuan Agricultural University, Chengdu, 611130 China; 2 Department of Veterinary Anatomy and Histology, Shaheed Benazir Bhutto University of Veterinary and Animal Sciences, Sakrand, Sindh 67210 Pakistan; 3 State Key Laboratory of AgroBiotechnology, China Agricultural University, Beijing, 100193 China; 4 National Engineering Laboratory for Animal Breeding, Key Laboratory of Animal Genetics and Breeding of the Ministry of Agriculture, Beijing Key Laboratory for Animal Genetic Improvement, College of Animal Science and Technology, China Agricultural University, Beijing, 100193 China

**Keywords:** Cell cycle, Cleavage rate, Melatonin, Metaphase II oocyte, Parthenogenetic activation, Vitrification

## Abstract

**Background:**

This study investigated the effect of melatonin (MT) on cell cycle (G1/S/G2/M) of parthenogenetic zygotes developed from vitrified-warmed mouse metaphase II (MII) oocytes and elucidated the potential mechanism of MT action in the first cleavage of embryos.

**Results:**

After vitrification and warming, oocytes were parthenogenetically activated (PA) and *in vitro* cultured (IVC). Then the spindle morphology and chromosome segregation in oocytes, the maternal mRNA levels of genes including *Miss, Doc1r, Setd2* and *Ythdf2* in activated oocytes, pronuclear formation, the S phase duration in zygotes, mitochondrial function at G1 phase, reactive oxygen species (ROS) level at S phase, DNA damage at G2 phase, early apoptosis in 2-cell embryos, cleavage and blastocyst formation rates were evaluated. The results indicated that the vitrification/warming procedures led to following perturbations 1) spindle abnormalities and chromosome misalignment, alteration of maternal mRNAs and delay in pronucleus formation, 2) decreased mitochondrial membrane potential (MMP) and lower adenosine triphosphate (ATP) levels, increased ROS production and DNA damage, G1/S and S/G2 phase transition delay, and delayed first cleavage, and 3) increased early apoptosis and lower levels of cleavage and blastocyst formation. Our results further revealed that such negative impacts of oocyte cryopreservation could be alleviated by supplementation of warming, recovery, PA and IVC media with 10^− 9^ mol/L MT before the embryos moved into the 2-cell stage of development.

**Conclusions:**

MT might promote cell cycle progression via regulation of MMP, ATP, ROS and maternal mRNA levels, potentially increasing the first cleavage of parthenogenetic zygotes developed from vitrified–warmed mouse oocytes and their subsequent development.

## Background

Oocyte cryopreservation, an adjunct to artificial assisted reproductive technologies, can provide medical assistance for women with ovarian cancer [1], premature ovarian failure [2] and those delaying the childbearing until later ages [3, 4]. It can also help build superior breeding pools for livestock [5, 6]. Live offspring can be obtained from the vitrified oocytes following warming, fertilization and embryo transfer [7]. However, the cleavage rates of embryos derived from the cryopreserved oocytes are significantly decreased after either *in vitro* fertilization [8, 9] or parthenogenetic activation [10, 11], thus potentially affecting their subsequent embryonic development.

During oocyte vitrification/warming, the meiotic spindle stability and integrity play very crucial role in completion of meiosis and further development after fertilization [12]. The proteins of MAPK-interacting and spindle-stabilizing protein (MISS) [13] and DOC1R [14], mitogen-activated protein kinase (MAPK) substrates are required for normal spindle formation and chromosome congression as well as microtubule stabilization and the second polar body extrusion [15]. Intriguingly, in events of abnormal levels of these two proteins following oocyte cryopreservation, the spindle morphology and chromosome alignment are likely to be disturbed. Such abnormal spindle morphology caused by oocyte cryopreservation [12, 16–18] usually results in abnormal chromosome segregation [19] and aneuploidy in oocytes and zygotes [20, 21], substantially affecting cleavage rate and subsequent embryonic development. After fertilization, the maternal mRNA degradation and translation are essentially required for maternal-to-zygotic transition and are regulated by transformation licensing factor BTG4 [22] and translation initiation factor EIF4E protein [23] respectively, before zygotic genome activation (ZGA) takes place. Both proteins can also co-regulate the maternal RNA degradation [23]. In addition, YTHDF2, a crucial reader to mediate the degradation of m6A-modified mRNAs [24, 25], and SEDT2, which can regulate the H3K36 trimethylation [26–28] are also required for ZGA. If YTHDF2 and SEDT2 or their mRNAs levels are fluctuated abnormally, the ZGA process can be interfered and thus affecting zygote development. It has been reported that oocyte cryopreservation can lead to down-regulation of mRNA levels [29], alterations of mRNA expression of genes related to maternal-to-zygote transition in 2-cell embryos [10] and 1575 differentially expressed genes in 2-cell embryos compared to those in the control group [30]. These alterations are believed to inevitably affect the quality of vitrified-warmed oocytes and their subsequent embryonic development. Therefore, it will be of great value to further elucidate the potential mechanism underlying the reduced developmental competence of vitrified-warmed oocytes after fertilization by investigating the mRNA levels of essential genes including *Miss, Doc1r, Btg4, Eif4e, Ythdf2* and *Setd2*.

After fertilization or parthenogenetic activation, a zygote is formed and its subsequent embryonic development is closely related to cell cycle progression [31–33]. The cell cycle is a four-stage process consisting of Gap 1 (G1), Synthesis (S), Gap 2 (G2) and mitosis (M) phases. It should be noted that in case when the duration or onset of zygotic cell cycle is initiated in an abnormal state, the developmental progression of embryos would be blocked. The longer duration of zygote S phase leads to a lower implantation rate [31]. The delay of S phase initiation or a shorter S phase can also reduce the blastocyst development rate [34]. In addition, the length of zygotic S phase is different between species or different strains/breeds of the same species. For instance, the length of the zygotic S phase in mice (AK, B6, CB and D2 strains), cattle and human was 6–11 h [35], 6.2–10.4 h [32] and 5.7–13.8 h [31], respectively. In our previous report we have shown that vitrification of mouse oocytes can led to the zygotic G1/S phase transition delay [36]. However, it is yet unclear whether the S phase delay is related to the ORC2 (a DNA replication licensing factor) [37] and if the duration of the S phase can be altered. It also remains to be elucidated whether there is relationship between the S phase initiation delay and zygotic cleavage rate.

During *in vitro* culture, melatonin (MT) has been reported to promote development of both fresh [38–43] and/or vitrified oocytes/embryos [10, 42, 44–46], mainly by one or more events such as modulating the oxidative stress, improving mitochondrial function, regulating spindle assembly, chromosome arrangement and expression of key genes, and inhibiting the apoptosis. Previously we have also reported that MT supplementation during the whole length of experiment (warming, recovery, parthenogenetic activation, and embryo culture steps) might promote G1-to-S progression via regulation of ROS, GSH and expression of cell cycle-related genes, potentially increasing the parthenogenetic development ability of vitrified–warmed mouse oocytes [10, 36]. Although the results of our previous reports [10, 36] have improved our understanding of MT implication in developmental competence of cryopreserved oocytes/embryos, it remains to be explored whether the cleavage and blastocyst formation rates can also be improved following MT supplementation before the embryos move into the 2-cell stage of development.

Therefore, with the foregoing question in mind, in the present study, we investigated the effect of MT on: 1) the cleavage rate of parthenogenetic zygotes from vitrified-warmed mouse oocytes and their subsequent development, 2) the mitochondrial function in G1 phase, ROS level in S phase, DNA damage level in G2 phase, and the duration and transition of S phase in zygotes, and 3) the pronuclear formation of vitrified-warmed oocytes after parthenogenetic activation by detecting the stability of spindle morphology and chromosome segregation in vitrified oocytes and the mRNA level of key maternal genes i.e., *Btg4, Orc2, Eif4e, Doc1r, Miss, Setd2 and Ythdf2* in activated oocytes.

## Material and methods

Unless otherwise indicated, all chemicals were purchased from Sigma-Aldrich (St. Louis, MO, USA). All experimental procedures were carried out in strict accordance to the regulations of the animal ethical and welfare committee (AEWC) of Sichuan Agricultural University China (approval code: AEWC2016, 6 January 2016).

### Oocyte collection

Outbred female ICR mice (aged 6 weeks) were purchased from Dashuo Company, Chengdu, China, and kept in sterilized cages under standard housing conditions: light controlled at 14:10 (06:00–20:00 light source), ambient temperature at 18–25 °C, and humidity at 50–70%. After a two-week adaptation period, female mice were induced to superovulate by an initial intraperitoneal injection of 10 IU equine chorionic gonadotropin (PMSG, Ningbo Shusheng Veterinary Drug Co., Ltd., Ningbo, China), followed by 10 IU human chorionic gonadotropin (hCG, Ningbo Shusheng Veterinary Drug Co., Ltd., Ningbo, China) 48 h later to induce ovulation. Female mice were euthanized by cervical dislocation. The cumulus-oocyte complexes (COCs) were collected from oviducts at 12–14 h after hCG treatment and recovered in M2 medium supplemented with 3 mg/mL bovine serum albumin. Cumulus cells were dispersed with 300 IU/mL hyaluronidase and then washed in M2 three times for subsequent experiments.

### Oocyte vitrification and warming

Oocytes were vitrified using an open-pulled straws (OPS) method as described in our previous study [10]. Briefly, the straws (0.25 mL; IMV, L’Aigle, France) were heat-softened and pulled manually to get straws of approximately 3 cm in length and 0.10 and 0.15 mm in inner and outer diameter, respectively.

Vitrification-warming procedures were carried out as per our laboratory practice [10]. Briefly, oocytes were first equilibrated in 10% ethylene glycol (EG) + 10% dimethyl sulfoxide (DMSO) for 30 s, then loaded into the narrow end of OPS with EDFS30 solution which consisted of DPBS medium containing 300 g/L Ficoll, 0.5 mol/L sucrose, and 20% FBS, 15% (v/v) EG and 15% (v/v) DMSO, for 25 s. Finally, the straws containing oocytes were plunged into liquid nitrogen quickly. During warming, oocytes were rinsed in 0.5 mol/L sucrose for 5 min, and then washed three times in M2 medium.

### Parthenogenetic activation (PA) and embryo culture

The methods for parthenogenetic activation of oocytes and the *in vitro* culture of resultant embryos were adopted as described previously [10]. Briefly, all oocytes in each group were recovered in M2 medium (recovery medium) for 1 h of culture in a CO_2_ incubator (Thermo Electron Corporation, Marietta, OH, USA) before activation. The activation medium was Ca^2+^-free human tubal fluid (HTF) supplemented with 10 mmol/L SrCl_2_. After washing three times in activation medium, oocytes were incubated first in activation medium for 2.5 h and then in regular HTF (Millipore, MR-070-D) without SrCl_2_ for 3.5 h at 37.5 °C in a humidified atmosphere with 5% CO_2_ in air. Both activation medium and HTF for subsequent short culture of oocytes were supplemented with 2 mg/mL cytochalasin D. Finally, oocytes were removed from above media and cultured in KSOM-AA medium (Millipore, MR-121-D). The 2-cell embryo and blastocyst rates were calculated at 24 h post activation (hpa) and 96 hpa. At 24 hpa, all the embryos in vitrification + MT group were transferred to KSOM-AA medium without MT. Melatonin (10^− 9^ mol/L) was added in warming, recovery (M2 medium), parthenogenetic activation, and embryo culture (KSOM-AA) media, whereas no MT was added in the Control and Vitrification groups during all stages of procedure.

The concentration of MT (10^− 9^ mol/L) used in the present study was adopted from our previous study [10] where we have reported that 10^− 9^ mol/L of MT concentration can improve the developmental competence of vitrified-warmed mouse M II oocytes. MT solution preparation (1 mol/L): 0.0237 g of MT (M5250–250 mg, Sigma,purity: 98%.) was dissolved in 100 μL of DMSO and then MT solution was fully mixed. Finally, the solution was evenly divided to 10 μL per tube and stored in a refrigerator at − 20 °C. Working solution: after multiple (proportional) dilutions, the working solution was obtained and used in experiments.

### Observation of pronuclear formation and zygotic cleavage

Pronuclear formation was observed every 1 h from 2 to 8 hpa using the stereoscopic microscope (SMZ1500, Nikon, Japan). To clearly show the pronucleus in bright field, a high resolution image (10 × 10) was captured using an advanced microscope (BX53, Olympus, Tokyo, Japan). Similarly, zygotic cleavage was observed every 1 h from 11 to 18 hpa under stereoscopic microscope. The first cleavage occurred shortly after the zygote nucleus is formed and it was completed when two blastomeres were apparent. Time for 10% of activated oocytes with (without) pronucleus or of 2-cell embryo formation was defined as the onset (end) of G1 phase or M phase, respectively. Similarly, the time for 50% of activated oocytes with (without) pronucleus or of 2-cell embryo formation was defined as mean time of onset (end) of G1 phase or M phase, respectively. The length of G1 or M phase = the time of end - the time of onset. The mean length of G1 or M phase = the mean time of end - the mean time of onset. Pronuclear formation rate = number of cells with pronucleus/number of parthenogenetic oocytes× 100%. Cleavage rate = number of 2-cell embryo/number of activated oocytes × 100%.

### Detection of zygotic mitochondrial membrane potential in activated oocytes

Mitochondria in zygotes were stained by the ΔΨm-specific probe JC-1. According to the manufacturer’s guidelines (Beijing Solarbio Science & Technology Co., Ltd., Beijing, China), JC-1 probe was diluted to final concentration of 10 μg/mL in M2 solution. All zygotes at 5 hpa were stained in a humidified incubator containing 5% CO_2_ at 37 °C for 15 min. The JC-1 reaction was conducted in darkness. Then the zygotes were washed three times for 5 min each in M2 solution without JC-1 probe. Finally, they were placed on a clean glass slide and photographed under a fluorescence microscope (BX53, Olympus, Tokyo, Japan). The fluorescence images were recorded as TIFF files using a built-in camera. Interpretation notes are as follows: High ΔΨm, JC-1 forms complexes known as J-aggregates. Low ΔΨm, JC-1 remains in the monomeric form. When excited at 510 nm, JC-1 monomers emit a green fluorescence with a maximum at ~ 527 nm. Aggregates of JC-1 emit an orange-red fluorescence with a maximum at ~ 590 nm. The ratio of red fluorescence to green fluorescence was recorded as mitochondrial membrane potential (Δψm) of oocytes. The intensity of red and green fluorescence in each oocyte was measured using Image J software (version 1.48; National Institutes of Health, Bethesda, MD).

### Detection of ATP content in zygotes

According to our previous report [42], zygotes at 5 hpa from each group were collected and processed for detection of ATP content. For this purpose, oocytes were initially washed three times with M2 and added to an Eppendorf tube containing 20 μL of ATP lysate for ATP detection (groups of 10 zygotes each.) ATP levels were determined according to the manufacturer’s instructions (A095–2, Nanjing Jiancheng Bioengineering Institute, Nanjing, China). Briefly, ten zygotes of each group were collected and transferred to lysis buffer of 20 μL for use. Then, the enzyme working solution was configured and samples were treated according to the instructions. ATP levels were measured using a multi-plate reader containing chemiluminescence (Varioskan LUX, Thermo, US). Sample ATP concentration was calculated using a standard curve generated from nine ATP gradient concentrations ranging from 0 mol/L to 2 μmol/L.

### Detection of S-phase cell cycle progression in zygotes

Zygotic S phase progress was checked by using the EdU assay kit (C10310–3, RiboBio, Guangzhou, China). At 5, 6, 7, 8, 9, 10, 11 and 12 hpa, zygotes were collected for EdU assay. The detailed operations followed the manufacturer’s guidelines of the EdU assay kit. Then, treated oocytes were transferred to clean slide containing VECTASHIELD mounting medium with DAPI and sealed with glass cover. Finally, slides with zygotes were checked under epifluorescence microscope (BX53, Olympus, Tokyo, Japan). The green fluorescence indicated EdU labeling. Zygotes with green fluorescence in the pronuclear area were labeled as being in the S phase. Time for 10% of zygotes with EdU labeling was defined as the time of onset (or end) of S phase; similarly, time for 50% of zygotes with EdU labeling was defined as mean time of onset (or end) of the S phase. The length of S phase = the time of end - the time of onset. The mean length of S phase = the mean time of end - the mean time of onset.

### Assessment of spindle morphology and chromosome segregation

The spindle morphology and chromosome segregation were assessed by using immunofluorescence assay. Briefly, oocytes of 0, 1 and 2 hpa were selected for assessment of spindle morphology and chromosome segregation. Oocytes were fixed in 4% (w/v) paraformaldehyde and then permeabilized in permeate (PBS containing 1% Triton X-100 (v/v) for 1 h at room temperature. After blocking of oocytes with 1% BSA for 1 h at room temperature, they were stained with FITC-anti-α-tubulin antibody (Sigma, F2168) at a dilution of 1:2,000 for 1 h at room temperature and washed three times for 5 min each in the wash buffer (PBS containing 0.01% Triton X-100 and 0.1% Tween 20). Then, treated oocytes were transferred to slide containing VECTASHIELD mounting medium with DAPI and sealed with glass cover. Finally, slides with zygotes were examined under epifluorescence microscope (BX53, Olympus, Tokyo, Japan). According to the previous study [12], spindle morphology was classified into grades 0–4. Grade 0 = no detectable spindle microtubules. Grade 1 = severely diminished spindle that is less than 50% of the normal spindle in size and asymmetry. Grade 2 = mildly diminished spindle that is slightly asymmetric in size on both sides. Grade 3 = equivalent to the normal spindle in size and shape. Grade 4 = spindle with excessive microtubule polymerization, namely, widening of the spindle poles, presence of astral microtubules emanating from the spindle poles. The criteria for chromosome segregation were defined as follows: The presence of two distinct sets of chromosomes in the cytoplasm was defined as chromosome segregation. If the spindle in the cytoplasm was consistent with the MII stage and the chromosomes were arranged on the equatorial plate, however, only one set of chromosomes was found in the cytoplasm, then they were defined as unseparated.

### Detection of ROS level, early apoptosis and DNA damage

The levels of ROS in zygotes of 9 hpa of each group were detected according to our laboratory’s protocol as described previously [10]. Briefly, for quantification of intracellular ROS levels, oocytes from each group were incubated (in dark) in M2 supplemented with 20 μmol/L 2, 7-dichlorodihydrofluorescein diacetate (H2DCFDA, Invitrogen, Carlsbad, CA, USA) for 30 min at 37 °C, washed three times in M2 medium containing 3 mg/mL bovine serum albumin, and then put into 20 μL droplets on a clean slide. Fluorescence images were recorded under epifluorescence microscope (BX53, Olympus, Tokyo, Japan).

Similarly, according to the manufacturer’s guidelines of Annexin-V kit (C1062L, Beyotime biotechnology, Shanghai, China), 2-cell embryos were collected and transferred to working solution of Annexin-V. After 30 min incubation at room temperature (~ 25 °C), zygotes were washed two times (5 min each) in PBS containing 3 mg/mL bovine serum albumin. In the end, treated embryo were transferred to slide containing VECTASHIELD mounting medium with DAPI and sealed with glass cover and checked under epifluorescence microscope (BX53, Olympus, Tokyo, Japan). The fluorescence intensities of ROS in zygotes and Annexin-V in 2-cell embryos were analyzed using Image J software (version 1.48; National Institutes of Health, Bethesda, MD).

DNA damage was analyzed by immunofluorescence assay, which was similar to spindle morphology analysis. Zygotes of 12 hpa were chosen for this test. Immunofluorescence was performed with the following antibodies: Rabbit anti-p-H2A.X (CST, 2577, p-Ser139, 1:800) and CoraLite594-conjugated Goat Anti-Rabbit IgG (Proteintech, SA00013–4, 1:200). Zygotes were incubated with primary antibody overnight at 4 °C, and then they were incubated with secondary antibody for 1 h at 37 °C. After incubation, zygotes were washed 3 times (5 min each). Finally, zygotes were transferred to slide with 20 μL M2 medium. Fluorescence images were recorded under epifluorescence microscope (BX53, Olympus, Tokyo, Japan). The fluorescence intensities of zygotes were analyzed using Image J software (version 1.48; National Institutes of Health, Bethesda, MD). The level of DNA damage showed positive correlation with the fluorescence intensity in the pronuclear area. The fluorescence intensity of gamma-H2A.X was calculated as following: Fluorescence intensity of gamma-H2A.X = total gamma-H2A.X fluorescence intensity of pronuclear/total pronuclear area. The total pronuclear area was chosen and calculated by freehand selection tool of Image J software. The stronger the fluorescence intensity was, the more serious the DNA damage.

### Quantitative polymerase chain reaction (Q-PCR)

The total cDNA was isolated from 2-hpa oocytes (≥20) using TransScript-Uni Cell to cDNA Synthesis SuperMix for Q-PCR kit (TransGen Biotech, Beijing, China). Then, cDNA was quantified by Q-PCR using a TransStart Tip Green qPCR SuperMix Kit (TransGen Biotech, Beijing, China) on a CFX Connect Real-Time Detection System (Bio-Rad, Hercules, CA, USA) under standard conditions. The cycle threshold (Ct) value used to calculate the relative expression was the average of three replicates and was normalized against that of the reference gene (*Gapdh*). The primer information is summarized in Table 1. The mRNA expression levels were calculated using the 2^-△△Ct^ method [47].

**Table 1:** Information of primers used in RT-qPCR

Genes	Accession ID	Primer Seq (5′→3′)	Product Length, bp	Tm, °C
*Btg4*	NM_019493.4	F: TGAACAACCCAAAGAGCGTCTACC	103	55
		R: AACCCACGACCATCTGCCAAATG		
*Orc2*	NM_001025378.2	F: TGATTCATGTCTTACGAAGCCT	107	55
		R: AGAAAGTCCAATGTAGGAAGGG		
*Eif4e*	NM_001313980.1	F: ACTTTTGGGCTCTATACAACCA	82	55
		R: ATCCCGTCCTTAAAAAGTGAGT		
*Miss*	NM_001045483.1	F: GTCCTTTAGGTACACAGGGATC	207	55
		R: GATATGACGCTTCAGGAGTAGG		
*Doc1r*	NM_026373.4	F:ACGGACCTGCTGTCTGTCATAGAG	146	55
		R: TTGCGTTCTGTCTCTGCCAAGC		
*Ythdf2*	NM_145393.4	F:TTGCCTCCACCTCCACCACAG	111	55
		R:CCCATTATGACCGAACCCACTGC		
*Setd2*	NM_001081340.2	F: GGAGGCAGACACGGAGACAGAG	139	55
		R: ATCTGGTGGCTCCTGGCTTCTC		
*Gapdh*	NM_008084.3	F: CATGGCCTTCCGTGTTCCTA	104	55
		R: GCCTGCTTACCACCTTCTT		

### Experimental design

**Fig. 1:**
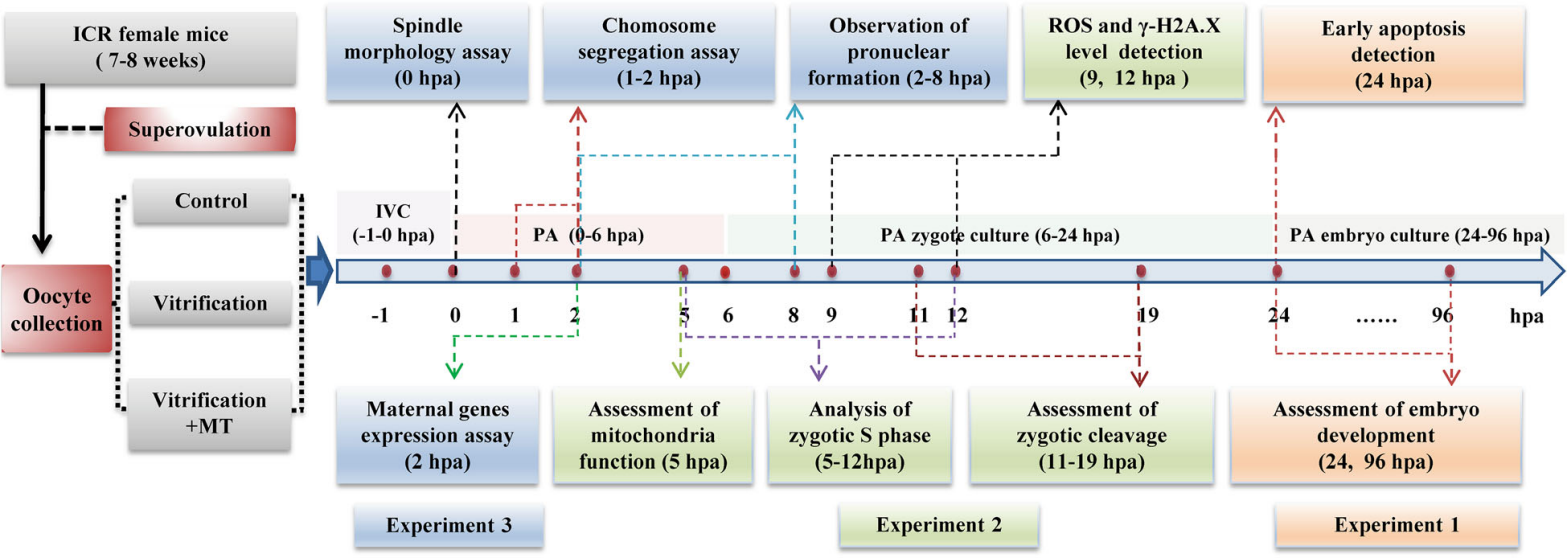
Flowchart of experimental design. Melatonin (10^− 9^ mol/L) was added in warming, PA and *in vitro* culture media before the embryos moved into the 2-cell stage of development (24 hpa). hpa: hours post-activation

The outline for the design of the experiments is presented in Fig. 1. This study was consisted of experiments 1, 2 and 3. In each of the experiments all the fresh oocytes were randomly divided into three groups: untreated (control), or vitrified by open-pulled straw method without (vitrification group) or with MT supplementation (vitrification + MT group). After vitrification/warming, the mouse MII oocytes were *in vitro* cultured for 1 h, followed by parthenogenetic activation of oocytes for 6 h and *in vitro* culture of resultant embryos for up to 90 h. The 10^− 9^ mol/L of MT was added in warming, recovery, PA and *in vitro* culture media and the treatment time of oocytes or zygotes in warming, recovery, PA and *in vitro* culture media was 5 min, 1 h, 6 h, and 18 h, respectively. After 24 hpa, 2-cell embryos in vitrification + MT group were transferred into *in vitro* culture medium without MT. The total time of MT treatment was 25 h and 5 min.

In experiment 1, impact of MT on cleavage of the parthenogenetic zygotes from vitrified-warmed oocytes and their subsequent development was investigated. Firstly, we observed the cleavage rate of activated oocytes and detected the early apoptosis in their resultant zygotes to investigate the influence of oocytes vitrification on the development of parthenogenetic zygotes. Then the 2-cell embryos were *in vitro* cultured for 72 h in KSOM-AA without MT supplementation and the blastocyst formation rate was assessed to explore whether supplementation of MT before embryos moved to 2-cell stage might improve their development to blastocysts.

In experiment 2, effect of MT on cell cycle progression during zygotic development in mice was investigated. Firstly, we observed the first cleavage of the parthenogenetic zygotes every 1 h under microscope during their *in vitro* development from 11 to 19 hpa. Then G1/S and S/G2 phase transition, and the S phase duration in zygotes at 5–12 hpa were determined by EdU staining. The mitochondrial membrane potential, ROS level and DNA damage in zygotes were detected at 5, 9 and 12 hpa, respectively.

In experiment 3, effect of MT on pronuclear formation after parthenogenetic activation of vitrified-warmed mouse oocytes was investigated. Firstly, pronuclear formation was observed under microscope every 1 h in activated oocytes at 2–8 hpa to investigate the potential role of MT in G1 phase zygotes. Then the spindle morphology in oocytes at 0 hpa and the chromosome segregation in activated oocytes at 1–2 hpa were analyzed by immunofluorescence assay. The levels of mRNA of key maternal genes (i.e., *Btg4、Orc2、Eif4e、Doc1r、Miss、Setd2* and *Ythdf2*) were detected in activated oocytes at 2 hpa by using RT-qPCR.

### Statistical analysis

Statistical analyses were performed by using one-way analysis of variance (ANOVA) followed by a post-hoc Fisher’s least significant difference (LSD) test using SPSS statistical software (v.20.0, IBM, Chicago, IL, USA). Mean times of these events corresponded to the moment at which half of the zygotes had pronucleus, entered/exited the S-phase, and cleaved, and were calculated from the equation of the regression lines. Data were analyzed by ANOVA after arcsine transformation for the percentages. The data are expressed as mean ± standard error. *P*-value < 0.05 was considered as statistically significant. All experiments were replicated at least three times.

## Results

### Melatonin promoted the first mitotic division of zygotes from vitrified-warmed mouse oocytes after parthenogenetic activation

**Fig. 2:**
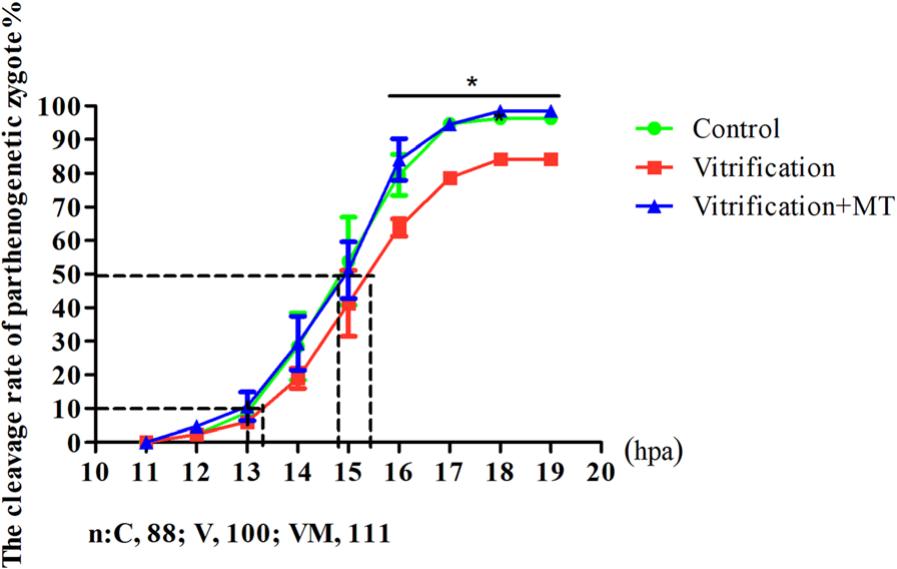
The first mitotic division of parthenogenetic zygotes. “*” and black line represent that the cleavage rate of the control and vitrification + MT groups are significantly different compared to vitrification group at 16–19 hpa under the black line (*P <* 0.05). Dotted lines represent the time when 10% or 50% of zygotes cleaved in each group, respectively. The data are expressed as mean ± standard error of three independent trials. “*n*” represents the cell number used in this experiment

As shown in the Table 2, the majority of the activated oocytes showed normal cleavage to 2-cell embryos. After oocyte vitrification, the cleavage rate of oocytes at 24 h post activation decreased significantly (*P* < 0.05) compared to the control group. During the *in vitro* culture of activated oocytes, 50% of them were calculated for early cleavage at 15.47 ± 0.14 h in vitrification group and at 14.76 ± 0.22 h in the control group post activation, respectively (Fig. 2), and it was observed that both groups differed significantly (*P* < 0.05), indicating that oocyte vitrification can induce a delay in the initiation of the first cleavage. When MT was added in vitrification group, the time for the first cleavage of half of the activated oocytes was significantly shortened (14.77 ± 0.18 hpa; *P* < 0.05) and the cleavage rate of oocytes at 24 hpa was significantly increased (*P* < 0.05). Interestingly, these rates were observed to be similar (*P* > 0.05) to those of the control group (Fig. 2 and Table 2), indicating that MT can promote the first cleavage of the vitrified-warmed oocytes after parthenogenetic activation. Then the 2-cell embryos were cultured *in vitro* for 72 h without MT supplementation, and it was noted that the blastocyst rate in vitrification group was significantly lower (*P* < 0.05) than that of the control group which was similar (*P* > 0.05) to that of Vitrification + MT group (Table 2), indicating that MT might potentially improve the quality of 2-cell embryos derived from the vitrified mouse oocytes when it was added before the activated oocytes moved to the 2-cell embryo stage.

**Table 2:** Effect of addition (phase-wise) of melatonin on parthenogenetic development of vitrified mouse MII oocytes

Groups	NO. of oocytes activated	No. of activated oocytes developed to		
		2-cell embryos, %	Blastocysts-1, %	Blastocysts-2, %
Control	84	79(94.40 ± 1.06)^a^	65(77.39 ± 1.68)^a^	65(81.97 ± 1.34)^a^
Vitrification	71	59(83.10 ± 0.96)^b^	37(53.02 ± 3.15)^b^	37(63.76 ± 3.46)^b^
Vitrification + MT	77	73(94.91 ± 0.28)^a^	58(76.47 ± 0.60)^a^	58(80.57 ± 0.69)^a^

The numbers of 2-cell embryos and blastocyst were counted at 24 and 96 h post-activation (hpa) respectively. The rates of 2-cell embryo and blastocysts-1 were calculated from the number of activated oocytes, the rates of blastocysts-2 were calculated from the number of 2-cell embryos. The values indicate the mean ± standard error (MSE) of three independent experiments. Values with different superscripts (a, b, and c) in the same column are significantly different (*P* < 0.05)

### Melatonin decreased early apoptosis of the 2-cell stage parthenogenetic embryos from vitrified-warmed oocytes

**Fig. 3:**
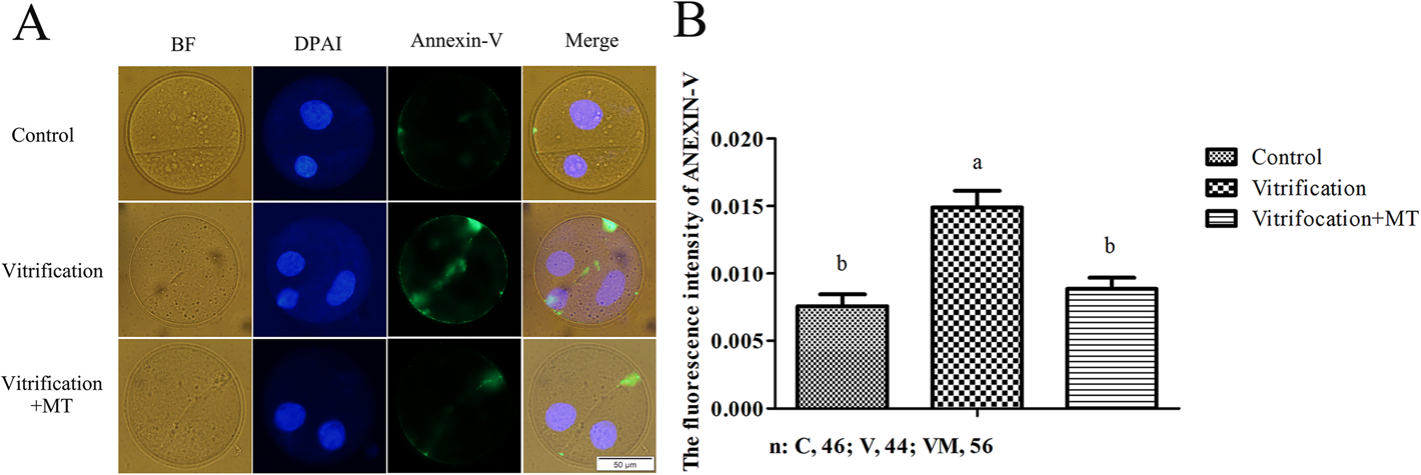
Annexin-V staining of 2-cell embryos. The Y-axis represents the average fluorescence intensity per unit area. The fluorescence intensity per unit area = the total fluorescence density of a single 2-cell embryo/ the total area of a single 2-cell embryo. The experiment was repeated three times. The data are expressed as mean value ± standard error. Values with different superscripts (**a**, **b**) are significantly different (*P <* 0.05). “*n*” represents the cell number used in this experiment

To explore whether the quality of parthenogenetic embryos might be affected and to elucidate the potential role MT may play in this process, we used the early apoptosis detection kit to identify early apoptotic level within the 2-cell embryos. As shown in Fig. 3, Annexin-V signals were observed on the cell membrane of the 2-cell embryos. The Annexin-V signal level in vitrification group (0.0149) was significantly higher (*P* < 0.05) than that of the control group (0.00758). After MT supplementation in vitrification group, Annexin-V level (0.00886) decreased significantly (*P* < 0.05) and recovered to the normal level of the control group (*P* > 0.05). These results suggested that MT may improve the quality of 2-cell embryos by decreasing their early apoptosis, thereby promoting their subsequent development to blastocysts.

### Melatonin ameliorated the oocyte vitrification-inflicted delay of zygotic S phase

**Fig. 4:**
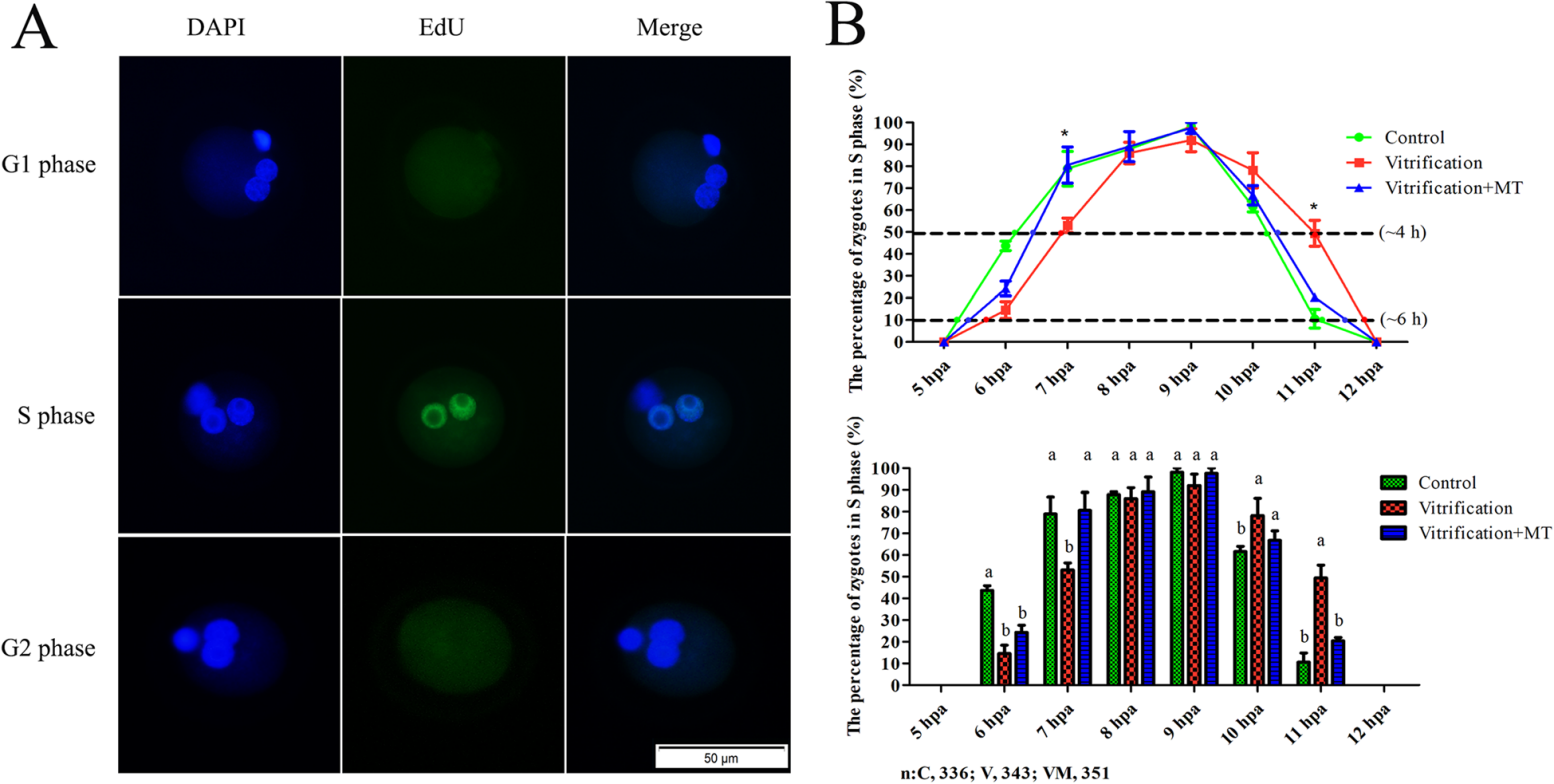
S phase transition of PA zygotes. **a** depicts zygotes with green fluorescence in pronuclear in the S phase. DAPI marks DNA. Scale bar = 50 μm. **b** represents a histogram depicting the proportion of S phase zygotes of each group at each time point. **c** is a broken line graph representing the proportion of S phase zygotes at each time point, which is used to reflect the length of S phase and time variation trend of each group in the S phase. The data are expressed as mean ± standard error of four independent trials. Values with different superscripts (a, b) are significantly different (*P <* 0.05)。“*n*” represents the cell number used in this experiment

As shown in Fig. 4b, the S phase cells at 6, 7 and 11 hpa after EdU labeling in parthenogenetic zygotes were 43.71 ± 2.18%, 78.92 ± 7.89% and 10.58 ± 4.19%, respectively in untreated control group. However, the S-phase fraction in vitrification group decreased to 14.56 ± 3.81% in zygotes at 6 hpa, and 53.11 ± 3.30% at 7 hpa, and increased to 49.46 ± 5.89% at 11 hpa. When MT was added in vitrification group, the S-phase fractions at 6, 7 and 11 hpa were 24.31 ± 3.34%, 80.63 ± 8.28% and 20.34 ± 1.61%, respectively. At 6 hpa, the data of vitrification group was similar to that of vitrification + MT group (14.56 ± 3.81% vs. 24.31 ± 3.34%, *P* > 0.05). In addition, the data of vitrification + MT group was significantly different from the control group (24.31 ± 3.34% vs. 43.71 ± 2.18%, *P* < 0.05). But at 7 and 11 hpa, statistical analysis revealed that the values of vitrification + MT group significantly differed from those of vitrification group (*P* < 0.05) and were similar to the corresponding control group (*P* > 0.05). These results indicated that the oocyte vitrification delayed the zygotic S phase transition (G1/S; S/G2) and that MT can alleviate vitrification-inflicted delay. However, the length of S phase, as defined by the time for more than either 10% or 50% of zygotes entering the S phase, remained stable for about 4 or 6 h in all three groups, indicating either oocyte vitrification or vitrification + MT treatment may not change the duration of S phase in their parthenogenetic zygotes.

### Melatonin can improve mitochondrial function in zygotes

**Fig. 5:**
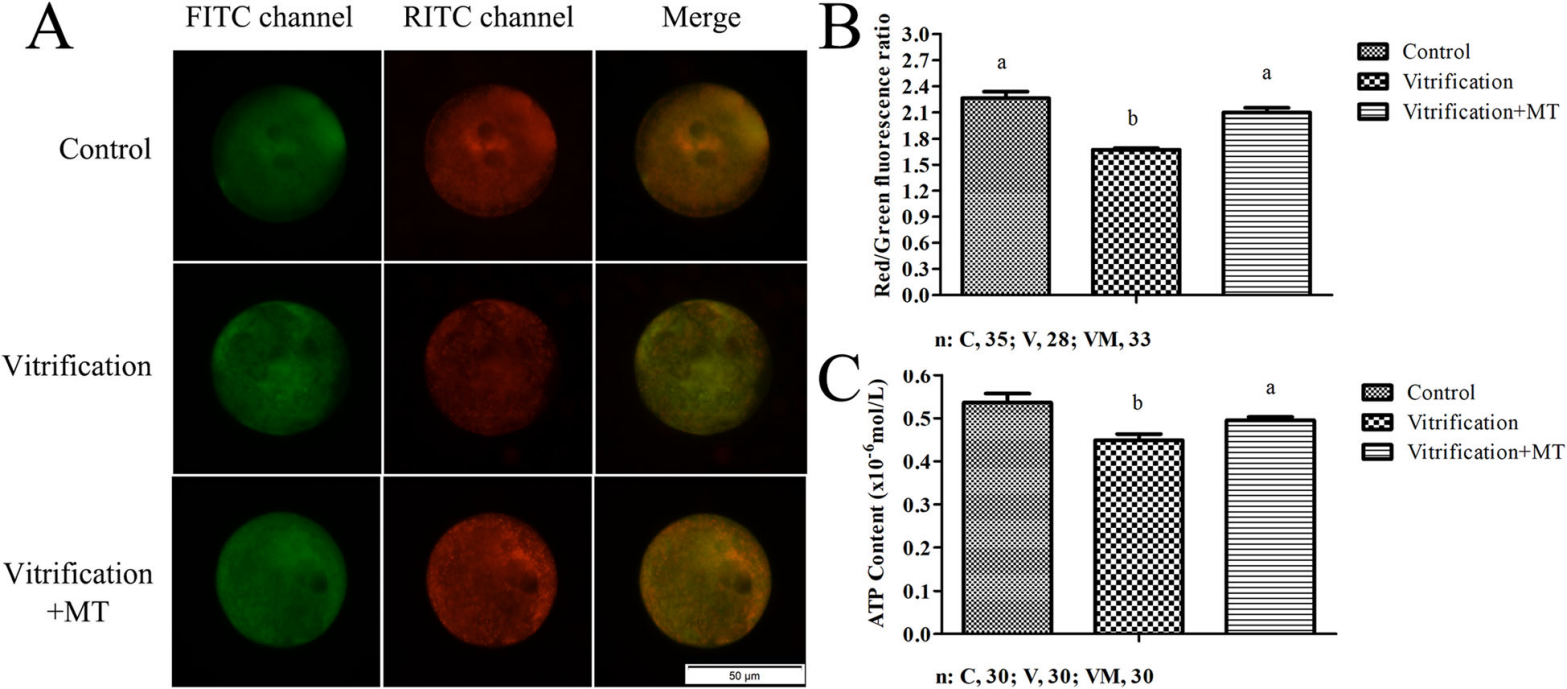
Mitochondrial membrane potential (MMP) and ATP contents of G1 zygotes. **a** depicts the state of MMP in each group. FITC channel represents that JC-1 is in the monomeric form, emitting a green fluorescence. RITC channel represents that JC-1 forms complexes emitting an orange-red fluorescence. The zygote is being in late G1 phase after 5 hpa. Scale bar = 50 μm. **b** shows the red/green fluorescence ratio of MMP between the groups. **c** shows the ATP content between the groups after 5 hpa. The data are expressed as mean ± standard error of four independent trials. Values with different superscripts are significantly different (*P <* 0.05). “*n*” represents the cell number used in this experiment

As shown in Fig. 5, when mouse oocytes were parthenogenetically activated (PA) for 5 h, the resultant zygotes at the late G1 PN stage showed significantly lower mitochondrial membrane potential (MMP) (Fig. 5b) and ATP levels (Fig. 5c) in vitrification group (1.67 ± 0.02 and 0.448 ± 0.015) than those of the control (2.26 ± 0.08 and 0.536 ± 0.021), respectively. When MT was added in the warming, recovery and PA media, the MMP (2.10 ± 0.06) and ATP levels (0.495 ± 0.007) in zygotes were significantly increased (*P* < 0.05) and were similar to those of the control group (*P* > 0.05). These results indicated that MT may promote zygotic G1/S transition potentially by modulating the zygotic mitochondrial function in G1 phase.

### Melatonin can alleviate excessive ROS and DNA damage induced by oocyte vitrification

**Fig. 6:**
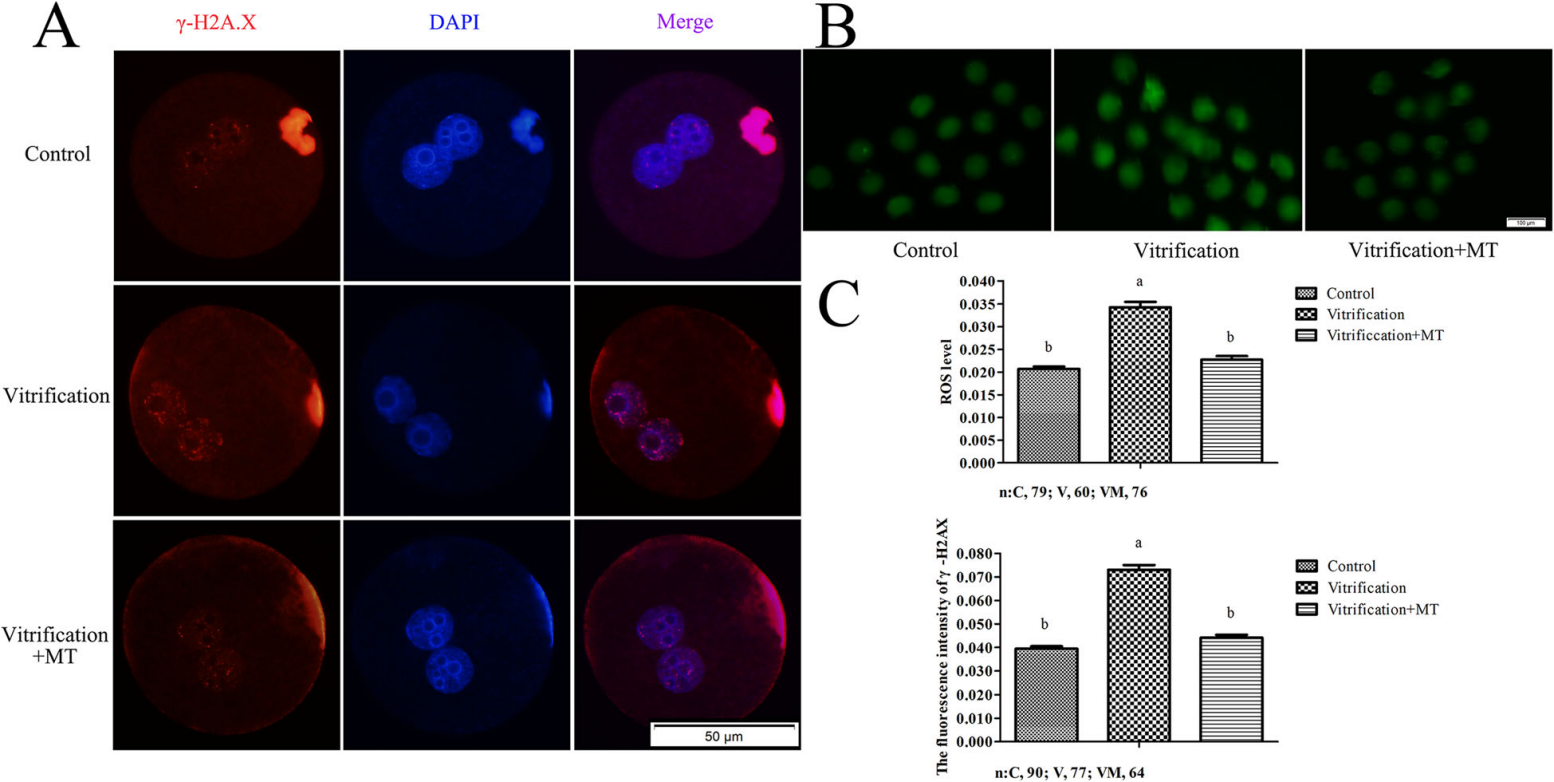
ROS and γ-H2A.X levels in zygotes. ROS (**b**) and γ-H2A.X levels (**a**) were assessed at 9 and 12 hpa respectively. The data are expressed as mean value ± standard error of three independent trails. Values with different superscripts (**a**, **b**) are significantly different (*P <* 0.05). “*n*” represents the cell number used in this experiment

As shown in Fig. 4b, at 9 hpa, more than 90% of the resultant parthenogenetic zygotes were observed at the S phase. At the same time point, ROS level in vitrification group (0.0343 ± 0.0012) was significantly higher (*P* < 0.05) than that of the control group (0.0207 ± 0.00052) which was similar (0.0227 ± 0.00078, *P* > 0.05) to vitrification + MT group (Fig. 6c). As shown in Fig. 6a, the phosphorylated H2A.X (γ-H2A.X) in pronuclei of zygotes at 12 hpa were stained red by immunofluorescence and their intensity served as an efficient marker for scoring of vitrification-induced DNA double-strand breaks (DSBs). The γ-H2A.X level in vitrification group (0.0730 ± 0.0021) was significantly higher (*P* < 0.05) than that of the control (0.0394 ± 0.0011) which was similar to vitrification + MT group (0.0441 ± 0.0012). These results indicated that the higher level ROS in S phase and DNA damage in G2 phase of zygotes due to oocyte vitrification can potentially be alleviated by MT supplementation.

### Melatonin promoted the formation of pronucleus in parthenogenetic zygotes from vitrified-warmed mouse oocytes

**Fig. 7:**
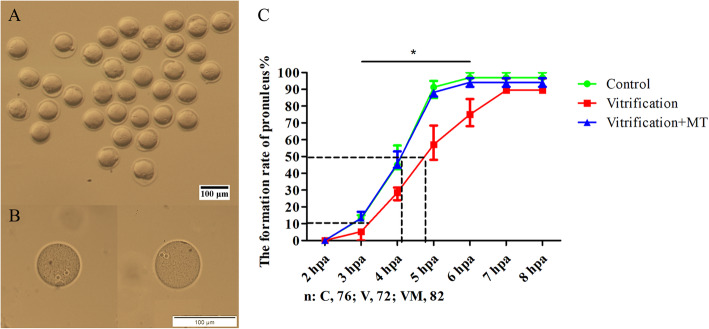
Pronuclear formation in PA zygotes. **a** was obtained by using stereomicroscope and original magnification of 100× was used for counting of pronuclear formation. **b** was obtained by using epifluorescence microscope and depicts the high resolution image of pronucleus. In **c**, the line chart depicts the rate of pronuclear formation from 2 to 8 hpa. “*” and black line represent that the control and vitrification +MT groups are significantly different compared to vitrification group at each time point under the black line (*P <* 0.05). Dotted line represents the time when 50% of oocytes formed pronuclear. The data are expressed as mean ± standard error of three independent trials. Scale bar = 100 μm. “*n*” represents the cell number used in this experiment

No pronuclear formation, as characterized by the appearance of nucleoli (Fig. 7b), was observed under the microscope at 2 hpa of oocytes. The pronucleus began to form at 3 hpa and reached the peak almost at 6 hpa (Fig. 7c). The pronuclear formation rate at 3–6 hpa in vitrification group (4.14 ± 2.14% to 75.74 ± 4.69%) was significantly lower than those of the control group (13.39 ± 0.85% to 97.54 ± 1.29%), indicating that the oocyte vitrification might delay the pronuclear formation in resulting zygotes. When MT was added in vitrification group, the pronuclear formation rate at 3–6 hpa was significantly (14.60 ± 1.27% to 94.07 ± 1.51%, *P* < 0.05) increased and was similar to (*P* > 0.05) that of the control group, indicating that MT can mitigate the delay of pronuclear formation following oocyte vitrification.

### Melatonin promoted the mitotic spindle stabilization in the vitrified-warmed oocytes

**Fig. 8:**
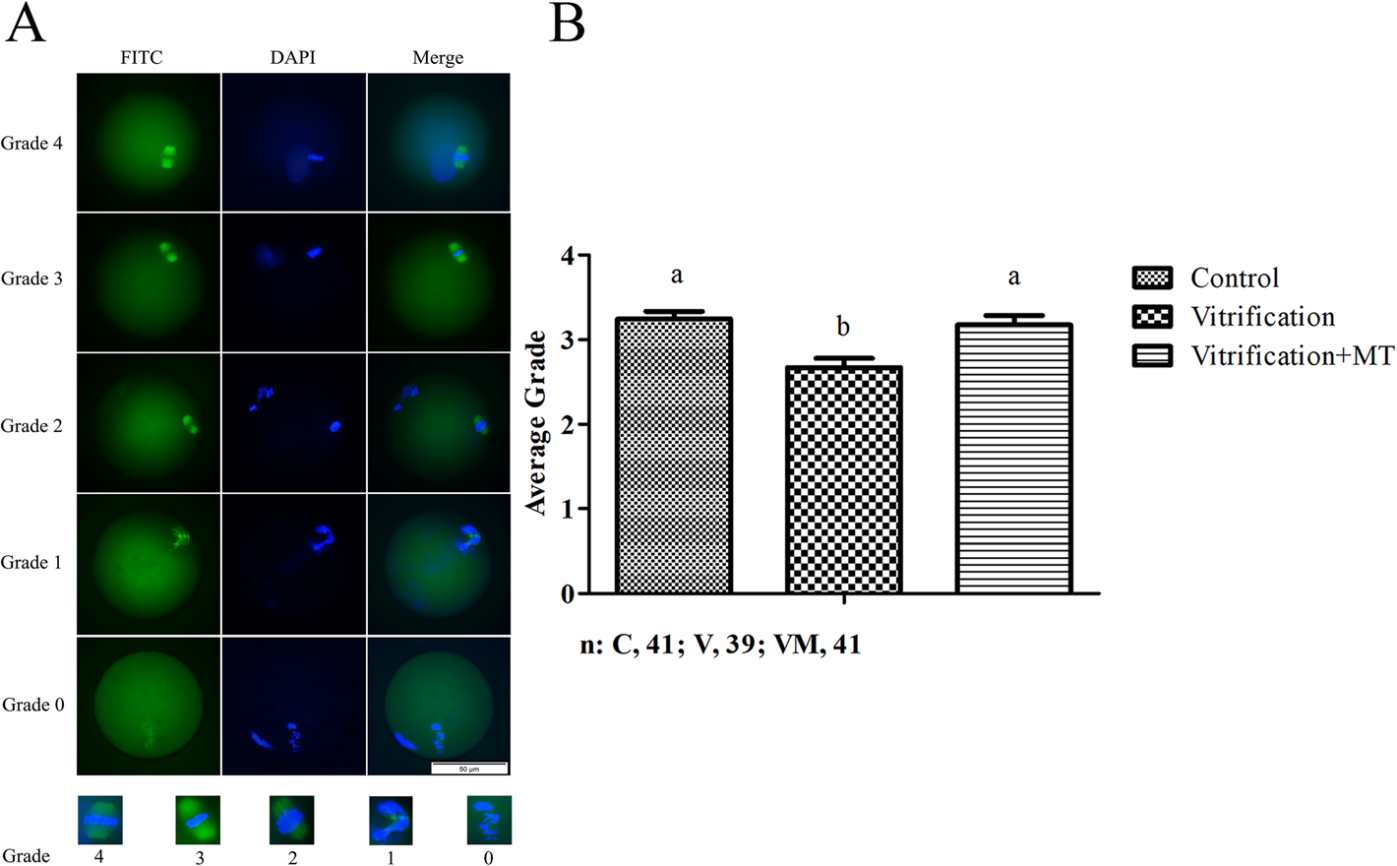
Morphology of spindle and chromosome after vitrification of MII oocytes. **a**, FITC represents spindle and DAPI represents DNA; **b** depicts the grade of spindle morphology between groups. The scale bar = 50 μm. The data are expressed as mean value ± standard error of four independent trails. Values with different superscripts (**a**, **b**) are significantly different (*P <* 0.05). “*n*” represents the cell number used in this experiment

As shown in Fig. 8a, the spindle configuration in oocytes was graded the higher the grade, more normal the oocytes were. The results of spindle morphology evaluation are depicted in Fig. 8b. Briefly, the average grade of spindle morphology in mouse MII oocytes was observed to be 3.24 ± 0.09 in the control group. However, following vitrification-warming and *in vitro* culture for 1 h, the average grade of spindle morphology of oocytes in vitrification + MT (10^− 9^ mol/L) group was significantly higher (3.17 ± 0.11 vs. 2.67 ± 0.11; *P* < 0.05) compared to the vitrified (without MT) group. Meanwhile, no significant difference was observed in the average score of spindle morphology between MT-treated and the fresh (control) groups, highlighting that MT supplementation might improve the spindle morphology in vitrified-warmed oocytes.

### Melatonin promoted chromosome segregation in vitrified-warmed oocyte after parthenogenetic activation

**Fig. 9:**
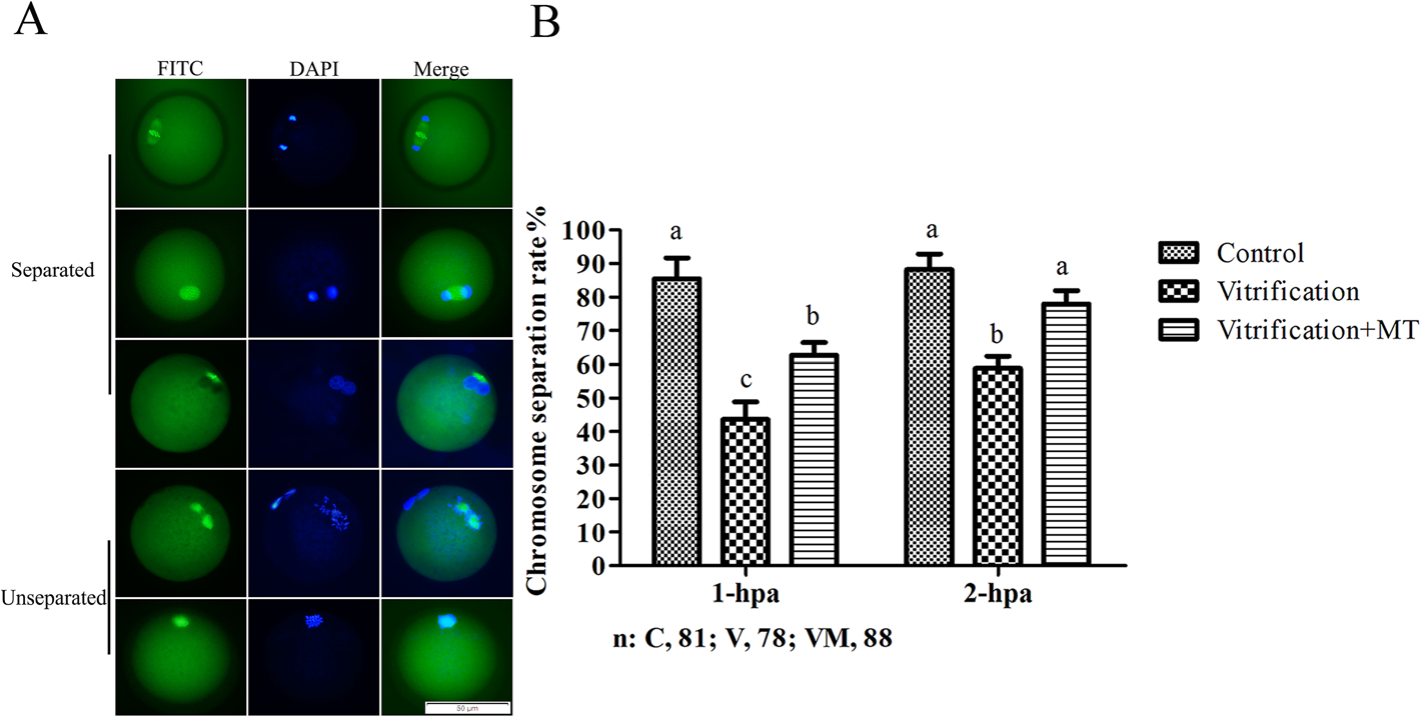
Chromosome segregation after PA of MII oocytes. **a**, with two apparent chromosome sets in oocyte cytoplasm represents that chromosomes are separated. If not, like the last two figures and images as shown in **a** represents that chromosomes remain unseparated. The scale bar = 50 μm. In **b**, histogram shows the rate of chromosome segregation at 1 and 2 hpa. The data are expressed as mean value ± standard error of four independent trails. Values with different superscripts ((a, b) are significantly different (*P <* 0.05). “*n*” represents the cell number used in this experiment

As shown in Fig. 9a, based on differences in the shape as observed under immunofluorescence, the chromosome segregation in oocytes was categorized into two subtypes/groups: separated and unseparated subtypes. Rates for all separated chromosome in oocytes were calculated and are given in Fig. 9b. The chromosome separation rates in oocytes at 1 and 2 hpa in vitrification group were 43.64 ± 5.28% and 58.90 ± 3.49%, respectively, and these rates were significantly lower (*P* < 0.05) than those of the corresponding controls (83.33 ± 6.25% and 85.70 ± 4.81%) and vitrification + MT groups (62.63 ± 3.79% and 77.93 ± 4.03%). Meanwhile, there was no significant difference (*P* > 0.05) in the chromosome separation rates at 2 hpa between the control and vitrification + MT groups. These results indicated that MT can promote chromosome segregation in the activated oocytes following vitrification.

### The protective role of melatonin against the effects of oocytes vitrification on the maternal mRNA level

**Fig. 10:**
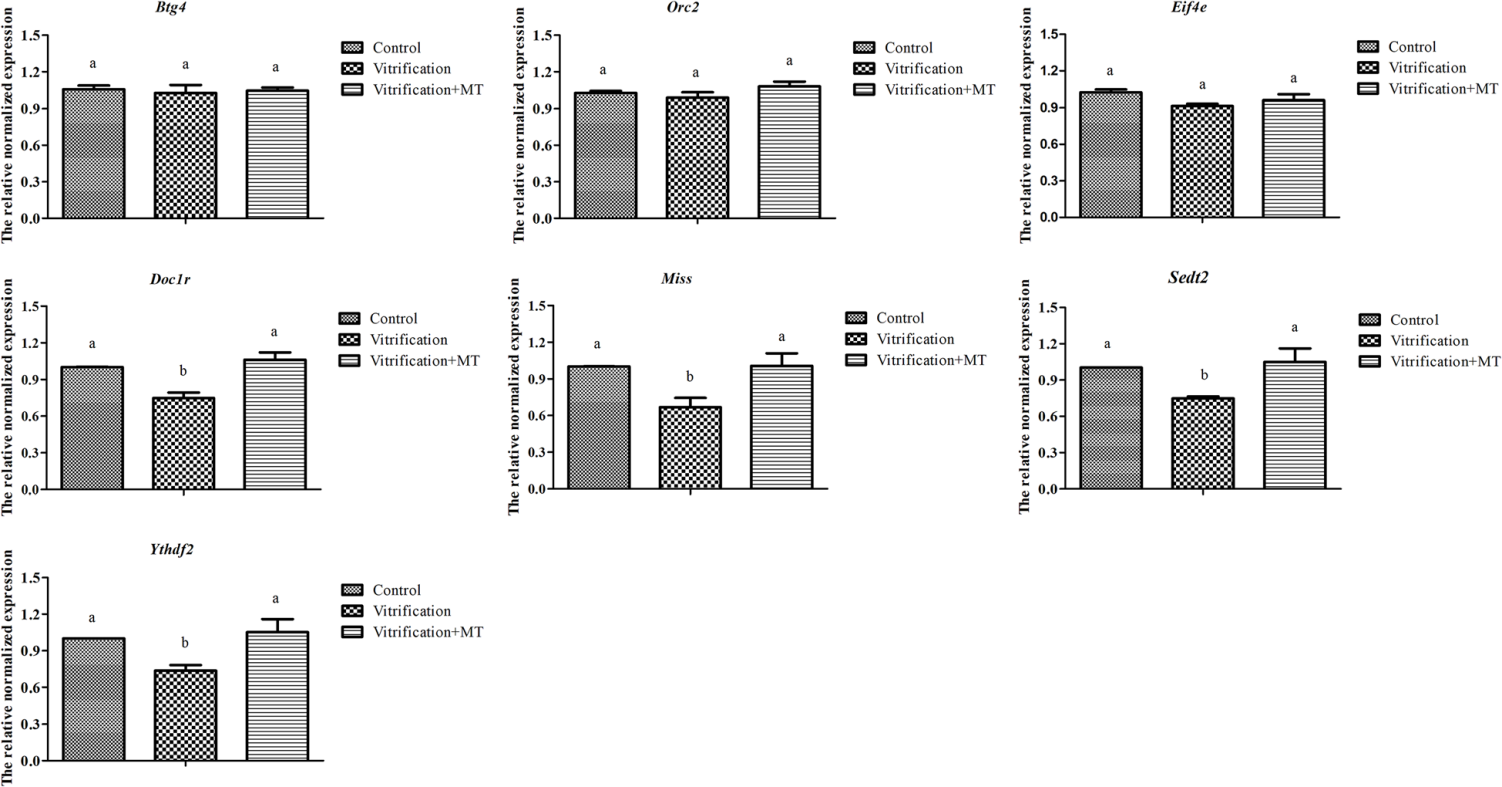
Maternal mRNA levels in oocytes at 2-hpa. The data are expressed as mean value ± standard error of three independent trails. Values with different superscripts (a, b) are significantly different (*P <* 0.05). Zygote number: control, 74, vitrification, 95, vitrification + MT: 95

As shown in Fig. 10, at 2 hpa of oocytes, the mRNA levels of maternal genes *Btg4*, *Eif4e,* and *Orc2* showed no significant difference (*P* > 0.05) between vitrification, vitrification + MT and the control groups. However, the mRNA levels of *Miss*, *Doc1r*, *Setd2* and *Ythdf2* genes in vitrification group were significantly decreased (*P* < 0.05) compared to the control group. Interestingly, when MT was added in vitrification group, the mRNA levels of these genes were significantly increased (*P* < 0.05) and recovered to the level of the control group (*P* > 0.05), indicating that MT can alleviate vitrification-induced adverse effects on the mRNA level of key maternally-derived genes in oocytes.

## Discussion

In this study, we observed the delayed first zygotic cleavage and higher early apoptosis level in 2-cell embryos developing from vitrified mouse MII oocytes, suggesting that vitrification can negatively impact the cell cycle in parthenogenetic zygotes and can lower their developmental potential. It is pertinent to mention that DNA replication occurs in the S phase of the cell cycle, and from zygote to adult, it is regarded as one of the most important events in reproduction, largely due to the fact that a single copy of the combined parental genome will be replicated an estimated five trillion times [48]. Therefore, the timing of DNA replication initiation and accurate replication is crucial for cellular propagation and survival. In our study, we found that the oocyte vitrification delayed the initiation (G1/S) and termination (S/G2) of DNA replication during zygotic S phase and was responsible for the lower cleavage rate. Intriguingly, this negative consequence was later alleviated when MT was added in warming, recovery (M2 medium), parthenogenetic activation, and embryo culture media before the activated oocytes moved into the 2-cell stage of their development. However, the vitrification (onset of oxidative stress condition) and MT treatment caused no significant impact on duration of the S phase. Interestingly, a similar phenomenon was observed in a study where it was shown that oxidative stress in the G1 phase zygotes treated with H_2_O_2_ (0.03 mmol/L) did not alter the S phase duration, but caused the delay of zygotic cleavage [49]. Although our understanding of such phenomenon is still incomplete, it appears that such finding might be related to the lack of a G1/S checkpoint in the oxidatively-damaged mouse zygotes [50].

In the present study, the G1/S phase transition was delayed in parthenogenetic zygotes following oocyte vitrification, indicating that the checkpoints or other such mediators may be present in the cell cycle at G1 phase (or events related to the duration of the G1 phase) and that might contribute to the disturbances in the cell to move into its next phase i.e., the S phase. It is well-established that the G1 phase acts as a preparatory phase where the cell synthesizes various enzymes and nutrients that are needed for DNA replication and cell division later on. In addition, such events also occur in the G1 phase as pronuclear formation, DNA and histone epigenetic modifications and synthesis of various proteins are required for zygote development [51]. ATP, a molecule mainly produced in the mitochondria, might be released to fuel the aforementioned cellular processes with a vital source of energy. Given that the mitochondrial function is linked to a number of biological events, we envisage that its dysfunction due to oocyte vitrification may be implicated in the G1/S phase transition delay in zygotes. In fact, oocyte cryopreservation has been reported to decrease the mitochondrial activity [42, 52–55] and MMP in the 2-PN embryos, leading to a lower ATP production. Interestingly, previously it has been shown that the up-regulation of glucose metabolism (with higher ATP production) during the male pronuclear formation can determine the early onset of the S phase in bovine zygotes [56]. Similarly, in the present study, the lower levels of ATP and MMP were observed in the G1 phase zygotes following oocyte vitrification. It is important to note that when MT was added, ATP and MMP levels recovered to the normal values and resulted in the acceleration of the G1 transition by approximately 0.8 h, potentially promoting the cleavage and blastocyst formation rates. We speculated that oocyte vitrification-induced mitochondrial dysfunction can potentially disturb biological events in the G1 phase of zygote and lead to delay of G1/S transition. In addition, it has been reported that MT is a versatile molecule that can mitigate and protect mitochondrial functions and dynamics by scavenging ROS, inhibiting the mitochondrial permeability transition pore and activating uncoupling proteins [57]. MT is believed to maintain the optimal mitochondrial membrane potential and preserves mitochondrial functions [57]. Therefore, based on our finding, it can be envisaged that MT can mitigate the mitochondrial dysfunction and can promote biological events of G1 phase and the G1/S phase transition. However, the precise underlying mechanism and implication of MT in promoting the G1/S transition by regulating mitochondrial functions remains to be further studied.

Simultaneously with a lower ATP production in the zygotes, the mitochondrial dysfunction was also characterized by abnormally increased ROS levels [58], leading to an exaggerated oxidative damage. It has been reported that oxidative stress-induced DNA damage can trigger the cell cycle checkpoints, leading to delay in the G2/M phase transition in zygotes about 2.5–3 h [49, 59]. It seems that the oxidative stress-induced DNA damage can activate the ATM/Chk1 and phosphorylate Cdc25 and Cdc2, which can further trigger G2/M arrest of zygote [49]. Furthermore, the oxidative stress-induced phosphorylation and activation of AMPK can inhibit CDK1 activity via p53/p21 pathways, thereby delaying the G2/M phase transition and facilitating the repair of DNA damage in zygotes [60]. Previously Lei and colleagues have shown that vitrification-inflicted mitochondrial oxidative damage can lead to the reduced developmental potential in vitrified mouse oocytes [61]. Interestingly, similar finding was also observed in the present study. We detected the mitochondria membrane potential at 5 hpa, ROS level at 9 hpa and γ-H2A.X at 12 hpa in zygotes, respectively and found that oocyte vitrification decreased the mitochondrial activity and increased ROS production and DNA damage, however, these damages were considerably alleviated by MT treatment, potentially promoting the cleavage rates. The oocyte vitrification-inflicted oxidative stress might delay or arrest the G2/M phase transition via activating ATM/Chk1 [49] or AMPK-p53/p21 [60] pathway, partially leading to delay or arrest of cleavage. It has been reported that MT is a powerful scavenger of ROS and can maintain the genome integrity [62]. Therefore, MT can be implicated in decreasing the DNA damage induced by ROS and preventing zygotes from arrest of G2/M transition.

In the present study, pronuclei were first formed between 2 and 3 h post oocyte activation and lasted about 4 h. However, the pronuclear formation rate was decreased significantly at 3 to 6 hpa when oocytes were vitrified-warmed followed by PA, indicating that the oocyte quality was affected considerably following vitrification. However, when MT was added in the vitrified-warmed oocytes, more pronuclei were formed at each time point. This similar finding was also observed in a previous study on aged oocytes where it was reported that the pronuclear formation rate at 5 h post *in vitro* fertilization of oocytes was significantly lower in the aged mouse (12 months, 35.5%) than in young counterparts (6–8 weeks, 88.1%) [63]. These authors further argued that this change in the rate of pronuclear formation may have resulted either due to the incorrect alignment of chromosome or premature chromosome segregation in the aged oocytes [63]. In addition, recently it was reported that after *in vitro* aging for 24 h, most of the mouse oocytes fragmented and no pronuclei were formed after intracytoplasmic sperm injection (ICSI), however, this situation was reversed in the aging + caffeine-treated group in which it was observed that 62.2% of oocytes produced pronuclei following ICSI and only 7.5% of oocytes led to fragmentation [64]. Therefore, above evidence indicates that the lower oocyte quality due to either vitrification or (*in vitro*) aging might contribute to the blockade of pronuclear formation to some extent, and the delay in the pronuclear formation can be alleviated either by MT or caffeine.

Although repolymerization of the meiotic spindle occurs in oocytes surviving the rigors of cryopreservation [12], it has been reported that the addition of certain agents such as docetaxel and paclitaxel (inhibitors of microtubule disassembly) [16] or MT [42] can stabilize the microtubules and reduce the spindle damage during cryopreservation. MT can stabilize the microtubules potentially by decreasing oxidative stress and regulating spindle assembly checkpoint [42]. Similarly, in the present study we observed that MT improved the average grades of spindle morphology (the higher grade it is, the lower spindle abnormality) in the vitrified-warmed oocytes after *in vitro* culture for 1 h, potentially promoting proper meiotic chromosome segregation. Although there is reasonable evidence that the protective effect of MT on spindle damage [42, 65–68] might be exhibited either by directly reducing the oxidative stress or through regulating spindle formation via its receptors [69, 70], however, the exact underlying mechanisms of MT action still remain to be clarified.

Maternal mRNA translation and degradation is crucial for the oocyte-to-zygote transition. A hallmark of this progress in mammals is its reliance on translation and the utilization of stored RNAs and proteins, rather than de novo transcription of genes, to sustain meiotic maturation and early embryonic development [23]. In these biological events, MAPK signal pathway is believed to play a pivotal role in regulating maternal mRNA translation and degradation [71]. After fertilization, MAPK activity is declined but does not completely inactivate until after pronuclear formation [72]. In this study, pronuclear formation was observed in oocytes at 3 hpa. Thus, in order to investigate the possible relationship of the MAPK cascade-related proteins with resultant pronuclear formation and subsequent embryo development, we analyzed their mRNA levels in oocytes at 2 hpa and found that the mRNA levels of *Eif4e* and *Btg4* did not change significantly in vitrification and vitrification + MT-treated groups. It seems that these two proteins might have been fully accumulated during oocyte maturation [73] and their further mRNA translation may not have occurred at this time point. Moreover, the mRNA level of *Orc2* (gene of DNA replication licensing factor) and the S phase duration (DNA replication period) also showed no significant differences between the groups, indicating that the oocyte vitrification and MT treatment might not affect the DNA replication period. However, we observed that the mRNA level of *Miss* and *Doc1r* were decreased significantly after oocyte vitrification. It can be envisaged that mRNAs (transcripts) of these genes might be translated to maintain the spindle and chromosome morphology and led to delay of the chromosome segregation and subsequent pronuclear formation, as these two MAPK substrates are reportedly implicated in either maintaining the spindle integrity or controlling the microtubule organization of MII mouse oocytes [15]. Similarly, in previous studies it has been reported that post-ovulatory aging of mouse oocytes leads to decreased level of MAD2 (a component of spindle assembly checkpoint) transcripts [74] but increases its (MAD2) protein level [75]. It is likely that the decreased mRNA levels of *Sedt2* and *Ythdf2* following oocyte vitrification might affect the early embryonic zygote-genome activation process, thus resulting in the lower development of embryos to blastocysts stage, as these two proteins are implicated in either regulation of the trimethylation modification of H3K36 [26–28] or degradation of methylated adenine maternal mRNA [24, 25]. This caveat is somehow supported by evidence from two previous studies where it was shown that when these two proteins were knocked out, embryo development was blocked in 1-cell [28] and 2-cell embryos [25]. Similarly, previous reports have indicated that cryopreservation can lead to an overall decrease of mRNA extracted from cryopreserved MII oocytes compared to the control group [29, 76]. Interestingly, a transcriptome-based analysis indicated that several downregulated genes were enriched in carbon metabolism, metabolic pathways, base excision repair, microtubule-based process, methylation, RNA transport, and cellular response to heat pathways and processes. In particular, some of the downregulated genes in vitrified oocytes were also enriched in the MAPK signaling pathway [76]. Given that the MAPK signaling pathway plays an important role in regulating the maternal mRNA translation and degradation [71], therefore, we believe that the MAPK signaling pathway might regulate translation or degradation of those key maternal mRNAs to mitigate the negative effects induced by vitrification. Interestingly, in the present study, we observed that after MT supplementation, the mRNA levels of key maternal genes i.e., *Miss*, *Doc1r*, *Sedt2* and *Ythdf2* were almost close to the control group. In previous studies, MT has been implicated in regulating various cellular and biological events in somatic cells [77] and oocytes [78] via MAPK signaling pathway. Hence, we speculate that MT might regulate the maternal mRNA levels in vitrified oocytes through MAPK signaling pathway. Since our understanding related to implication of MT in these biological events is still incomplete, therefore further focused studies are desired to provide concrete evidence in this regard.

## Conclusion

In summary, the findings from the present study indicate that the addition of MT (10^− 9^ mol/L) during warming, recovery, parthenogenetic activation of oocytes and *in vitro* culture of embryos (total 25 h 5 min of MT treatment) improved the developmental competence of vitrified-warmed oocytes. These ameliorative effects were observed as follows: 1) MT promoted the formation of pronuclei in PA zygotes via reducing the spindle damage in vitrified-warmed oocytes, potentially regulating the mRNA levels of key maternal genes (*Miss* and *Doc1r*) in activated oocytes. 2) MT promoted the first cleavage of zygotes potentially by regulating the G1/S and S/G2 phase transition via stabilization of the mitochondrial function and mitigation of oxidative DNA-damage. 3) MT partially improved the development of zygote to blastocyst via regulating ZGA related maternal mRNA levels (*Sedt2* and *Ythdf2*) and reduction of early apoptosis.

## Data Availability

All data generated or analysed during this study are included in this published article.

## Abbreviations

MT: Melatonin
MII: Metaphase II
ROS: Reactive oxygen species
ATP: Adenosine triphosphate
MISS: MAPK-interacting and spindle-stabilizing protein
MAPK: Mitogen-activated protein kinase
PA: Parthenogenetically activated
IVC: *In vitro* cultured
MMP: Mitochondrial membrane potential
ZGA: Zygotic genome activation
AEWC: Animal ethical and welfare committee
PMSG: Equine chorionic gonadotropin
hCG: Human chorionic gonadotropin
COCs: Cumulus-oocyte complexes
OPS: Open-pulled straws
EG: Ethylene glycol
DMSO: Dimethyl sulfoxide
EDFS30: DPBS medium containing 30% (v/v) ethylene glycol, 21% (w/v) Ficoll 70, and 0.35 mol/L sucrose
V: Volume
W: Weight
HTF: Human tubal fluid
KSOM-AA: Simple optimization of medium contained K ions and amino acid
hpa: Hours post activation
H2DCFDA: 2, 7-dichlorodihydrofluorescein diacetate
